# Nonparametric Clustering of Mixed Data Using Modified Chi-Squared Tests

**DOI:** 10.3390/e24121749

**Published:** 2022-11-29

**Authors:** Yawen Xu, Xin Gao, Xiaogang Wang

**Affiliations:** Department of Mathematics and Statistics, York University, Toronto, ON M3J 1P3, Canada

**Keywords:** clustering, mixed data, non-parametric, weighted chi-squared test

## Abstract

We propose a non-parametric method to cluster mixed data containing both continuous and discrete random variables. The product space of the continuous and discrete sample space is transformed into a new product space based on adaptive quantization on the continuous part. Detection of cluster patterns on the product space is determined locally by using a weighted modified chi-squared test. Our algorithm does not require any user input since the number of clusters is determined automatically by data. Simulation studies and real data analysis results show that our proposed method outperforms the benchmark method, AutoClass, in various settings.

## 1. Introduction

Mixed data that contain both continuous and discrete data are abundant in scientific research, especially in medical or biological studies. An effective clustering method for mixed data should partition a large complex data set into homogeneous subgroups that are manageable in statistical inference. Clustering methods thus have a wide range of applications in almost all scientific studies including financial risk analysis, genetic analysis, and medical studies. They are essential tools in analyzing large data sets.

Most of the clustering methods in the literature have been mainly focused on either continuous data or categorical data alone. The K-means algorithm has been widely used in industrial applications for a long time. Detailed descriptions and discussions can be found in Kaufman and Rousseeuw (2009) [1]. Non-Euclidean distances such as the Manhattan distance or Mahoblis distance have also been used. Model-based clustering methods for continuous data have been proposed in the literature, see for example Banfield and Raftery (1993) [2]. One of the most prominent methods in parametric clustering based on a mixture model is proposed by Bradley et al. (1998) [3]. The number of clusters and outliers can be handled simultaneously by the mixture model. Fraley and Raftery (1998) [4] propose choosing the number of clusters automatically using the model-based clustering method. For clustering categorical data, there are far fewer reliable methods. K-modes algorithm has been proposed by Huang (1998) [5] to extend the K-means to clustering categorical data. The AutoClass method proposed by Cheeseman and Stutz (1996) [6] is a well-known method in clustering. The AutoClass takes a data set containing both real and discrete-valued attributes and automatically computes the number of clusters and group memberships. This method has been used by NASA and helped to find infra-red stars in the IRAS Low-Resolution Spectral catalog and discovery classes of proteins (Cheeseman and Stutz 1996 [6]).

In clustering mixed data, the main difficulty lies in the fact that continuous and categorical sample spaces are intrinsically different. Although both can be made into metric spaces, the continuous sample space resides on a differentiable manifold while the categorical one is defined entirely on a lattice. Attempts have been made in the literature to combine the two spaces by using a global and general distance function (Ahmad and Dey 2007 [7]). This naive approach ignores the fact that the two sample spaces are topologically incompatible. Another approach is to apply different clustering algorithms to the continuous and categorical portions separately and combine the results. This approach, however, would sever the intrinsic connection between the continuous and categorical parts of one record. Each record is often assigned to different clusters for the continuous and categorical parts. It is often hard to reconcile this except by expanding the total number of clustering. Not only does this produce a larger than necessary number of clusters for the entire data sets, but a true cluster is also often found being split across many small clusters and renders the results to be very inaccurate. Alternatively, AutoClass combines information across probability spaces. However, the effectiveness of AutoClass depends on the validity of the assumed parametric model. Zhang et al. (2006) [8] showed that both K-modes and AutoClass do not perform very well when applied to benchmark categorical data sets from the UCI machine learning depository. Therefore, there is a need for a non-parametric clustering method for mixed data.

We extend the work by Zhang et al. (2006) [8] to cluster mixed data by using adaptive quantization of the continuous sample space. The quantization process was developed in the 1950s and it partitions the sample space through a discrete-valued map (Gersho and Gray 1992) [9]. For univariate cases, quantization is known as vector quantization and it is the fundamental process for converting analog signals or information into digital forms (Gersho and Gray, 1992) [9]. It has been used in studying pricing in finance as well as engineering. Theoretical properties of quantization in probability distributions can be found in Graf and Luschgy (2000) [10]. The process of clustering mixed data is then performed on the quantized product space. The key idea is inspired by the fact that any manifold can be locally modeled by a Euclidian space. Therefore, each neighborhood in the transformed product space can be locally characterized as a fine grid endowed with a Hamming Distance. The Hamming Distance is widely used in information and coding theory (Roman 1992 [11]; Laboulias et al., 2002 [12]). The statistical significance of a detected cluster is determined by a weighted local Chi-squared test. The advantage of our proposed method over AutoClass is demonstrated in simulations and by using two benchmark data sets from the UCI machine learning depository.

This paper is organized as follows. The method is proposed in Section 2. The clustering algorithm is presented in Section 3. Simulation results are provided in Section 4.

## 2. Clustering Methodology

In this section, we introduce quantization of the mixed sample space on which we adopt the Hamming Distance function to measure the relative positions of two data points. We also define a distance vector and an optimal separation point which are essential to measuring spatial patterns as well as the size of any detected clusters. Separation points are introduced to extract detected cluster patterns.

### 2.1. Joint Sample Space of Mixed Data

Consider a general data structure for a mixed data set with *p* nominal categorical attributes and *q* continuous attributes. The categorical sample space is defined on $\Omega_{p}=R^{p}$ while the continuous one is defined on $\Omega_{q}$. The product space for mixed data is then defined on the product space $\Omega_{p}⊗\Omega_{q}$. The sample size is denoted by *n*.

The categorical part of mixed data is represented by $X=(X_{i}^{j})$, with i=1,2,⋯,n and j=1,⋯,p. Furthermore, row and column vectors in the categorical portion are denoted by $X_{i}^{\left[\cdot \right]}$ and $X_{\left[\cdot \right]}^{j}$. The $j^{th}$ categorical attribute is categorized by $m_{j}$ levels defined by set $A_{j}=\left(a_{j1},\cdots ,a_{jm_{j}}\right),j=1,\cdots ,p$.

We denote the continuous part of a mixed sample with size *n* by $Z=(Z_{i}^{k})$, with i=1,2,⋯,n and k=1,⋯,q. Furthermore, we denote the row and column vectors in the categorical portion by $Z_{i}^{\left[\cdot \right]}$ and $Z_{\left[\cdot \right]}^{k}$. The $k^{th}$ attribute is a continuous random variable.

### 2.2. Quantization of Continuous Sample Space

Continuous data and discrete data are fundamentally different. Although the description provided by the continuous portion can be very detailed, it could carry excessive information that is not important for the clustering purpose. Furthermore, any pattern derived from the categorical part is based on a much coarse topology than the continuous counterpart. Since it is impossible to define a meaningful and objective manifold from a coarse data structure, the continuous one then must be mapped into a grid that is compatible with the relatively coarse topology from the categorical one.

The quantization is achieved in two steps. Firstly for observed realization $z_{i}^{j}$, continuous data are mapped onto the unit interval between 0 to 1 by applying the following formula:

$${\tilde{z}}_{i}^{k}=\frac{z_{i}^{k}-z_{min}^{k}}{z_{max}^{k}-z_{min}^{k}},\;\;\;\;k=1,\ldots ,q;i=1,\ldots ,n,$$

where $z_{min}^{k}$ and $z_{max}^{k}$ represent the minimum and maximum values of the *k* column. Secondly, for the standardized observations, the continuous random variable is then mapped or quantized into a discrete random variable with *M* levels in the following way:

$$Q\left({\tilde{z}}_{i}^{k}\right)=m,\;\;\;\;if\;\;\;\;\;\left(m-1\right)/M\leq {\tilde{z}}_{i}^{k}<m/M,$$

where m=1,2,⋯,M, where *M* can be any positive integer value. Different numerical values of *M* could have an impact on the quality of quantization and consequently the clustering result. A finer quantization grid might not be useful and could be more computationally intensive than a coarse one.

The number of levels *M* can be difficult to specify by a user with no prior information. Thus, we propose to choose level *M* adaptively by using *F* statistics based on the clustering results.

For any fixed numerical value of *M*, we perform an ANOVA test by treating each cluster as a separate group by using the generated clustering memberships. The F-statistic associated with the ANOVA test is recorded for different values of *M*. We then set the optimal numerical value for *M* by selecting the value that corresponds to the largest F-statistic computed before. Numerical results of the quantization level will be illustrated in Section 4.1.

### 2.3. Distance Vectors on Quantized Product Space

We use Hamming Distance (HD) to measure the relative separation of two categorical data points. To be more specific, for any two positions in the categorical sample space $\Omega_{p}$, $Q_{h}^{\left[\cdot \right]}=\left(Q_{h}^{\left[1\right]},\cdots ,Q_{h}^{\left[p\right]}\right)$ and $Q_{i}^{\left[\cdot \right]}=\left(Q_{i}^{\left[1\right]},\cdots ,Q_{i}^{\left[p\right]}\right)$, the HD between $Q_{h}^{\left[j\right]}$ and $Q_{i}^{\left[j\right]}$ on the *j*th attribute is

$$d\left(Q_{h}^{j},Q_{i}^{j}\right)=\left\{\begin{array}{cccc} \; & 0 & if & Q_{h}^{j}=Q_{i}^{j},\\ \; & 1 & if & Q_{h}^{j}\neq Q_{i}^{j}. \end{array}\right.$$

Further, we define the distance between the two positions, that is, the summation of the distance from each pair of the components. Therefore, we have the following:

$$HD\left(Q_{h}^{\left[\cdot \right]},Q_{i}^{\left[\cdot \right]}\right)=\sum_{j=1}^{p}d\left(Q_{h}^{j},Q_{i}^{j}\right).$$

After quantization, the new product space now resides on a high-dimensional grid. Since for a grid, there is no natural origin. We can define a reference point (S,T) in the quantized product space with $S=\left(s_{1},\cdots ,s_{p}\right)\in R^{p}$ and $T=\left(t_{1},\cdots ,t_{q}\right)\in R^{q}$. For the categorical portion, $HD_{C}\left(X_{i},S\right)$ can take values ranging from 0 to *p*; and for quantized continuous data, we have $HD_{Q}\left(Z_{i},T\right)$ can take values ranging from 0 to *q*.

We then define the Distance Vector (DV) based on Hamming distance for the categorical and quantized continuous portion, respectively. We define two individual vectors to record the frequencies of each categorical and quantized continuous distance value accordingly, that is, a (p+1)-element vector $DV_{C}\left(S\right)$ for the categorical data and a (q+1)-element vector $DV_{Q}\left(T\right)$ for the quantized part. To be more specific, $DV_{C}$ is defined as

$$DV_{C}\left(S\right)=\left(DV_{C}^{\left[0\right]}\left(S\right),DV_{C}^{\left[1\right]}\left(S\right),\cdots ,DV_{C}^{\left[p\right]}\left(S\right)\right),$$

and $DV_{Q}$ is defined as

$$DV_{Q}\left(T\right)=\left(DV_{Q}^{\left[0\right]}\left(T\right),DV_{Q}^{\left[1\right]}\left(T\right),\cdots ,DV_{Q}^{\left[q\right]}\left(T\right)\right).$$

The $j^{th}$ component in $DV_{C}$ and $h^{th}$ component in $DV_{Q}$ are given as the following:

$$DV_{C}^{\left[j\right]}\left(S\right)=\sum_{i=1}^{n}I\;\left[HD_{C}\left(X_{i}^{\left[\cdot \right]},S\right)=j\right],\;\;\;j=0,1,\cdots ,p;$$

$$DV_{Q}^{\left[h\right]}\left(T\right)=\sum_{i=1}^{n}I\;\left[HD_{Q}\left(Q_{i}^{\left[\cdot \right]},T\right)=h\right],\;\;\;h=0,1,\cdots ,q;$$

where I(A) is an indicator function that takes value 1 when event *A* happens and 0 otherwise.

If there is no cluster pattern at all, we would expect a uniform distribution of all possible cases. Then it is equally likely for a randomly chosen data point to take any possible position in the joint sample space. The DV vectors under uniform distribution are referred to as a *uniform* distance vector (UDV). Thus, a UDV records the expected frequencies under the null hypothesis that there are no clustering patterns in the data. Let **X** be a categorical portion of data and **Z** be a continuous portion of the data from a sample of size *n*, with each observation having an equal probability of locating at any position on space $\Omega_{p}⊗\Omega_{q}$. The expected value of DV and DV associated with the null hypothesis is denoted by $UDV_{C}$, $U=(U_{0},\cdots ,U_{p})$ for categorical data and $UDV_{Q}$, $V=(V_{0},\cdots ,V_{q})$ for continuous data, respectively.

Zhang et al. (2006) [8] provide the exact form of $UDV_{C}=\frac{n}{M_{1}}U^{*}$, where $M_{1}=\prod_{j=1}^{p}m_{j},j=1,2,\cdots ,p$; $m_{j}$ is the number of states in set $A_{j}$ for the $j^{th}$ attribute; and $U^{*}=\left(U_{0}^{*},U_{1}^{*},\cdots ,U_{p}^{*}\right)$ with

$$\begin{array}{c} U_{0}^{*}=1;\\ U_{1}^{*}=\left(m_{1}-1\right)+\left(m_{2}-1\right)+\cdots +\left(m_{p}-1\right);\\ U_{2}^{*}=\sum_{i<j}^{p}\left(m_{i}-1\right)\left(m_{j}-1\right);\\ \vdots \\ U_{p}^{*}=\left(m_{1}-1\right)\left(m_{2}-1\right)\cdots \left(m_{p}-1\right). \end{array}$$

Similarly, we obtain the exact form of the $UDV_{Q}$ for the quantized continuous part of the data. $UDV_{Q}=\frac{n}{M_{2}}V^{*}$, where $M_{2}=\prod_{j=1}^{q}l_{j},j=1,2,\cdots ,q$; $l_{j}$ is the the number of levels of quantization for the $j^{th}$ continuous attribute; and $V^{*}=\left(V_{0}^{*},V_{1}^{*},\cdots ,V_{q}^{*}\right)$ with

$$\begin{array}{c} V_{0}^{*}=1;\\ V_{1}^{*}=\left(l_{1}-1\right)+\left(l_{2}-1\right)+\cdots +\left(l_{q}-1\right);\\ V_{2}^{*}=\sum_{i<j}^{q}\left(l_{i}-1\right)\left(l_{j}-1\right);\\ \vdots \\ V_{q}^{*}=\left(l_{1}-1\right)\left(l_{2}-1\right)\cdots \left(l_{p}-1\right). \end{array}$$

### 2.4. Optimal Separation Point

If the initial starting point is chosen to be the center of one particular cluster, then the frequency of HD should demonstrate a decreasing pattern in a local region as the HD function records the frequency of data points from the center of the cluster and outwards. Small local bumps at the beginning part of the HD curve are expected if the initial starting point deviates slightly from the cluster center. The recorded frequencies might increase afterward when the function begins to record distances from another cluster. Therefore, the valley area indicates a natural place to separate one cluster from the rest. Separation points are, therefore, defined for this identification purpose.

Assume that the categorical data **X** and quantized continuous data **Z** are not uniformly distributed in the sample space $\Omega_{p}⊗\Omega_{q}$. Let $DV_{C}\left(S\right)=(DV_{C}^{\left[0\right]}\left(S\right),DV_{C}^{\left[1\right]}\left(S\right),\cdots ,$$DV_{C}^{\left[p\right]}{\left(S\right))}^{T}$, $S\in \Omega_{p}$ be the collection of all (p+1)-element $DV_{C}$ in the space $\Omega_{p}$ and $DV_{Q}\left(T\right)=(DV_{Q}^{\left[0\right]}\left(T\right),DV_{Q}^{\left[1\right]}\left(T\right)$, $\cdots ,DV_{Q}^{\left[q\right]}{\left(T\right))}^{T}$, $T\in \Omega_{q}$ be the collection of all (q+1)-element $DV_{Q}$ in the space $\Omega_{q}$, and let $U={\left(U_{0},U_{1},\cdots ,U_{p}\right)}^{T}$ be the $DV_{C}$ vector and $V={\left(V_{0},V_{1},\cdots ,V_{q}\right)}^{T}$ be the $DV_{Q}$ vector defined in the previous subsection. For a given distance value $j_{C},j_{C}=0,1,\cdots ,p$, for categorical distance values and $j_{Q},j_{Q}=0,1,\cdots ,q$, for quantized continuous distance values, there always exists at least one position ($S,T)\in \Omega_{p}⊗\Omega_{q}$, such that the frequency at this distance value is larger than the corresponding component, $U_{j}$ of the $UDV_{C}$ vector and $V_{j}$ of the $UDV_{Q}$ vector.

In order to proceed to a comparison between $DV_{C}$ and $UDV_{C}$ and between $DV_{Q}$ and $UDV_{C}$, we introduce a selection criterion for an optimal cut-off $r^{*}$. The categorical cut-off point was defined and proved by Zhang et al. (2006) [8]. Because our quantized continuous data behaves as categorical data, we extend that concept to a quantized portion of the data. If the cluster structure is present, the early segment of a $DV_{C}$ and $DV_{Q}$ with respect to a data center should contain substantially larger frequencies than the corresponding frequencies of the $UDV_{C}$ vector and $UDV_{Q}$ vector. Therefore, the range corresponding frequencies of the $UDV_{V}$ vector and $UDV_{Q}$ a vector that is consistently larger than the $UDV_{C}$ vector and $UDV_{Q}$ vector gives a reasonable indication of the *r*. This leads to an optimal $r_{C}^{*}$ for the categorical portion of data:

$$r_{C}^{*}\left(S\right)=\min_{j_{C}>0}\left\{j_{C}|\frac{DV_{C}^{\left[j_{C}\right]}\left(S\right)}{U_{j_{C}}}<1\right\}-1,S\in \Omega_{p}.$$

Similarly, optimal $r_{Q}^{*}$ for the quantized portion of data be:

$$r_{Q}^{*}\left(T\right)=\min_{j_{Q}>1}\left\{j_{Q}|\frac{DV_{Q}^{\left[j_{Q}\right]}\left(T\right)}{V_{j_{Q}}}<1\right\}-1,T\in \Omega_{q}.$$

The two quantities are used to identify relatively dense regions in the space of mixed data to help us to extract clusters accurately. Zhang et al. (2006) [8] gave a detailed explanation of the tuition of radius which is the maximum distance of the data points in this cluster to its center.

## 3. Algorithm

There are two key parts of the algorithm. Firstly, we detect whether there exist any statistically significant clustering patterns. We propose a weighted local Chi-squared test to determine if the observed distance vectors differ significantly from the uniform distance vectors associated with no cluster pattern. Secondly, if the patterns are significant, we further extract the clusters based on the optimal separation strategies described in the previous section.

We consider the null hypothesis $H_{0}$: There is no clustering pattern in the data set. The weighted local Chi-squared test statistic $\chi_{w}^{2*}\left(S,T\right)$ is defined as:

$$\begin{array}{cc} \chi_{w}^{2*}\left(S,T\right) & =\left(\frac{1}{p}+\frac{1}{q}\right)\left[\frac{1}{p}\chi_{C}^{2*}\left(S\right)+\frac{1}{q}\chi_{Q}^{2*}\left(T\right)\right]\\ \; & =\frac{pq}{p+q}\frac{1}{p}\chi_{C}^{2*}\left(S\right)+\frac{pq}{p+q}\frac{1}{q}\chi_{Q}^{2*}\left(T\right)\\ \; & =\frac{q}{p+q}\chi_{C}^{2*}\left(S\right)+\frac{p}{p+q}\chi_{Q}^{2*}\left(T\right),\;\;\;\;\;\;\left(S,T\right)\in \Omega_{p⊗q} \end{array}$$

The weighted local Chi-squared statistic $\chi_{w}^{2*}\left(S,T\right)$ is constructed to address the issue of an unequal number of variables for the continuous and categorical parts. We expect that a large number of variables tend to produce a large numerical value for the modified $\chi_{C}^{2*}$ and $\chi_{Q}^{2*}$. Therefore, each modified Chi-squared statistic is normalized by its corresponding number of variables for the categorical and continuous parts respectively. To ensure the total of the two weights to equal to 1, we further divide the sum of two normalized modified Chi-squares by the total of the two weights which equals 1/p+1/q.

Where the categorical part $\chi_{C}^{2*}\left(S\right)$ takes form as:

(1) $$\chi_{C}^{2*}\left(S\right)=\sum_{j=0}^{r_{C}^{*}}\frac{{\left(DV_{C}^{\left[j\right]}\left(S\right)-U_{j}\right)}^{2}}{U_{j}}+\frac{{\left(\sum_{j=0}^{r_{C}^{*}}DV_{C}^{\left[j\right]}\left(S\right)-\sum_{j=0}^{r_{C}^{*}}U_{j}\right)}^{2}}{\sum_{j=r_{C}^{*}+1}^{p}U_{j}},$$

and the quantized continuous part $\chi_{Q}^{2*}\left(T\right)$ takes the form:

$$\chi_{Q}^{2*}\left(T\right)=\sum_{j=1}^{r_{Q}^{*}}\frac{{\left(DV_{Q}^{\left[j\right]}\left(T\right)-V_{j}\right)}^{2}}{V_{j}}+\frac{{\left(\sum_{j=1}^{r_{Q}^{*}}DV_{Q}^{\left[j\right]}\left(T\right)-\sum_{j=1}^{r_{Q}^{*}}V_{j}\right)}^{2}}{\sum_{j=r_{Q}^{*}+1}^{q}V_{j}},$$

where *p* and *q* are the numbers of attributes from categorical and continuous data, respectively.

If the detected pattern passes a statistical test, we then proceed to extract a cluster by determining the cluster center ***C*** and estimating cluster radius ***R*** for mixed data. Therefore, a cluster center C is chosen where the $\chi_{w}^{2}$ has the maximum value. It is chosen to be:

$$C=\underset{\left(S,T\right)}{arg max}\chi_{w}^{2}.$$

Zhang et al. (2006) [8] gave the definition of radius which is the maximum distance of the data points in this cluster to its center. Radius is the distance at which the DV has its very first local minimum. Therefore, it is defined categorical Radius $R_{C}\left(C\right)$ as:

$$R_{C}\left(C\right)=\underset{0<j<p_{C}}{\mathrm{mim}}\left\{j|DV_{C}^{\left[j\right]}\left(C\right)<\mathrm{mim}\left(DV_{C}^{\left[j-1\right]}\left(C\right),DV_{C}^{\left[j+1\right]}\left(C\right)\right)\right\}-1.$$

For the quantized continuous part of the data, the optimal cut-off point is used as the quantized continuous radius $R_{Q}\left(C\right)$.

The step-by-step guide to our method is

**Step** **1.** For each position ***S***, we calculate HD in the categorical data; further, we obtain $DV_{C}$.
**Step** **2.** Standardize the continuous data and quantize the standardized data at a selected level. For each position calculate Hamming distance for quantized continuous data to obtain $DV_{Q}$.
**Step** **3.** Compare $DV_{C}$, $DV_{Q}$ with corresponding expected values $UDV_{C}$ and $UDV_{Q}$.
**Step** **4.** Determine cut-off points $r_{C}^{*}\left(S\right)$ and $r_{Q}^{*}\left(T\right)$ for categorical and quantized continuous data respectively; and further calculate the corresponding modified Chi-squared statistic $\chi_{C}^{2*}\left(S\right)$ and $\chi_{Q}^{2*}\left(T\right)$ and obtain the weighted local chi-square test statistic
  $$\chi_{w}^{2*}\left(S,T\right)=\frac{q}{p+q}\chi_{C}^{2*}\left(S\right)+\frac{p}{p+q}\chi_{Q}^{2*}\left(T\right).$$
**Step** **5.** Corresponding to the weighted local Chi-squared test, select the largest test statistic $\chi_{w}^{2*}\left(S,T\right)$; compare it with critical value $\chi_{\left(0.05\right)}^{2*}$ at the right tail. If the max ($\chi_{w}^{2*}\left(S,T\right)$) is smaller than $\chi_{\left(0.05\right)}^{2*}$, stop the algorithm; otherwise, continue to step 6.
**Step** **6.** Assign the position that has the largest test statistic $\chi_{w}^{2*}\left(S,T\right)$ as a center. Categorical data and continuous data share the same center position but with their own data points.
**Step** **7.** Calculate categorical a radius $R_{C}$ and continuous radius $R_{Q}$; label all data points within a radius in the cluster; record corresponding $\chi_{C}^{2*}\left(S\right)$ and $\chi_{Q}^{2*}\left(T\right)$; remove them from the current data set.
**Step** **8.** Repeat Steps 1 to 6 until no more significant clusters are detected.
**Step** **9.** Prune the membership assignment by calculating the minimum distance from each data point to center positions; If the membership is assigned differently to categorical data and continuous data, we further compare their p-values which are calculated from $\chi_{C}^{2*}\left(S\right)$ and $\chi_{Q}^{2*}\left(S\right)$; Re-assign the membership to the one with the larger p-value by the one with the smaller *p*-value.
**Step** **10.** Compute F-test statistic to choose the best-quantized level and corresponding clustering results as the final results.

## 4. Results

We conduct simulation studies and real data analysis to examine the performance of our proposed method. Classification rates and information gains are calculated to compare the performance of our proposed method with AutoClass.

### 4.1. Simulation Studies

In this section, we compare our method with AutoClass under various simulation settings. The simulation results are shown in Table 1, Table 2, Table 3 and Table 4. All attributes are generated independently. The simulation setting is as the following:

1. Set the number of categorical attributes p=10 and each attribute takes $m_{j}$ levels which are randomly selected from the set {4,5,6}; Set the number of continuous attributes q=9.
2. Set the number of clusters $K_{C}=K_{Q}=3$ or $K_{C}=K_{Q}=5$. The 3 cluster centers $C_{k}$ are denoted as $C_{k}=\left(c_{k,1},\cdots ,c_{k,10}\right)$, k=1,⋯,3. The 5 cluster centers $C_{k}$ are denoted as $C_{k}=\left(c_{k,1},\cdots ,c_{k,10}\right)$, k=1,⋯,3. For categorical centers, ensure the Hamming distance between any two of the centers is at least great than 5. For the continuous portion of data, choose a set of cluster means as 2, 8, and 16 for 3 clusters, or 2, 8, 16, 20, and 35 for 5 clusters;
3. Set sample size N=200 with cluster size $n_{1}=130$, $n_{2}=45$, and $n_{3}=25$; or set sample size N=1000 with the cluster size $n_{1}=500$, $n_{2}=200$, $n_{3}=100$, $n_{4}=100$, and $n_{5}=100$; or set sample size N=10,000 with the cluster size $n_{1}=5500$, $n_{2}=3000$, $n_{3}=1500$;
4. For categorical data, in the $k^{th}$ cluster with center $C_{k}$, generate $n_{k}$ 10-attributes vectors independently. More specifically, generate for each attribute from a multinomial distribution with a center probability of 0.7 and the rest probabilities are identically equal to $0.3/(m_{j}-1)$; For continuous data, $n_{k}$ 9-attributes vectors are 9 independent normal random variables with $\mu =C_{k}$ and $\sigma^{2}$ ranging from 0.25,0.5 and 1, respectively.

In our numerical results, the average classification rate (CR) and information gain (IG) rate with their corresponding standard deviations are used to evaluate the method’s performance. The CR measures the accuracy of an algorithm to assign data points to correct clusters. With given K clusters, the CR is defined by

$$CR\left(K\right)=\sum_{k=1}^{K}\frac{{\tilde{n}}_{k}}{n},$$

where *n* is the total number of data points and $\tilde{n_{k}}$ is the number of data points that have been correctly assigned to cluster *k* by an algorithm. Obviously, 0≤CR(K)≤1, and a larger CR(K) value indicates better performance of clustering. The information gain is an alternative criterion for assessing the performance of the clustering algorithm. It is the so-called cluster purity proposed by Bradley et al. (1998) [3]. Cluster purity essentially measures the information gain, which is the difference between the total entropy and weighted entropy for a given data partition, namely

informationgain(IG(K))=totalentropy−weightedentropy(K),

where the weighted entropy is calculated by

$$weighted\;entropy\left(K\right)=\sum_{k=1}^{K}\frac{n_{k}}{n}\times cluster\;entropy\left(k\right),$$

with

$$cluster\;entropy=-\sum_{l=1}^{L}\frac{{\tilde{n}}_{l}^{k}}{n_{k}}\log_{2}\left\{\frac{{\tilde{n}}_{l}^{k}}{n_{k}}\right\},$$

where ${\tilde{n}}_{l}^{k}$ is the number of data points with true label *l* in cluster *k*, $n_{k}$ is the number of data points known in cluster *k*, and *L* is the known number of classes. In this chapter, we take a ratio of IG(K)/total entropy, named information gain rate (IGR), which is similar to the classification rate between 0 to 1. It is necessary to point out that in some situations, the information gained may lead to misleading. For example, in our simulation studies, IG may be equal to 1 which means perfect clustering. However, indeed, it splits each true cluster into two clusters which is a wrong classification. This misleading situation happens in Table 1 and Table 2.

Table 1 shows the selection of quantization levels for a continuous portion of the data. As mentioned in Section 2.2, we use the largest F values to choose the selected quantization level which gives the best classification rate. Table 2, Table 3 and Table 4 provide results from simulated data with various settings of different sample sizes, number of clusters, and cluster sizes. The number of replications is 500 for Table 2 and Table 3, and 100 for Table 4. Table 2 is obtained by analyzing simulated data with a sample size of 200 with 3 clusters of the sizes of 130, 45, and 25. Simulated data for Table 3 has a sample size of 1000 and the number of clusters is 5, and each cluster size is 500, 200, 100, 100, and 100, respectively. Table 4 provides results from simulated data having a sample size of 10,000 with 3 clusters and each cluster size of 5500, 3000, and 1500, respectively.

As shown by Table 2, Table 3 and Table 4, our proposed algorithm consistently has a higher classification rate in comparison with that from AutoClass in all three different settings. For the three chosen settings, the mean classification rates, and information gain rates of the two algorithms are getting closer to each other and could even be identical. Table 3 shows us that our algorithm has higher IG rates compared to AutoClass. In Table 2 and Table 4, our algorithm has IG rates varying from 0.8923 to 0.93333. Although AutoClass could achieve one in some cases, this does not imply a perfect clustering because AutoClass tends to split each true cluster into unnecessary more clusters. Hence, overall, all tables show us that our algorithm has better performance in terms of CR and IGR by comparing it to AutoClass.

### 4.2. Real Data Analysis

We apply our method on to three real data sets. The first two data sets can be downloaded from the Machine Learning Repository website. One is Heart Data Set and the other one is the Australian Credit Approval Data Set. The third data set is collected by the RAND center at the University of Michigan.

Heart Data and Australian Credit Approval Data are downloaded from the Machine Learning Depository at the University of California at Irvine. Heart data contains 7 categorical, 6 continuous attributes, and 270 observations. The data provided the memberships for each observation. There are 2 clusters, absence, and presence. The cluster sizes are 120 and 150, respectively. In the Australian Credit Approval Data Set, there are 8 categorical attributes and 6 continuous attributes. The data set contains two clusters positive or negative with the corresponding cluster sizes 307 and 383. We compared our method with AutoClass. Table 5 shows the results from these two real data sets. From the table, we can tell that our method correctly identified the number of clusters for both data sets, while, AutoClass could not detect correct cluster numbers. In addition, our method has a higher classification rate compared to AutoClass. Our method has a classification rate of 81.48% for Heart data and 73.62% for Credit data. However, AutoClass has 44.44% and 52.71%.

We apply our proposed method to the health and retirement study (HRS) data set. Information about health, financial situation, family structure, and health factors was collected by the RAND center at the University of Michigan. We focus on the analysis of the status of depression depicted in the data set. Depression among children and adolescents is common but frequently unrecognized. The clinical spectrum of depression can range from simple sadness to major depressive disorders. A depression diagnosis is often difficult to make because clinical depression can manifest in so many different ways. Observable or behavioral symptoms of clinical depression may be minimal despite a person’s mental turmoil. The general population can then be partitioned naturally into two groups: depressed individuals and not depression people. We choose this scenario as the third test case for our clustering algorithm and compare its performance of ours with AutoClass.

We perform clustering based on six health factors: Smoking, Restless Sleep, High Blood Pressure, Frequent Vigorous Physical Activity, Difficulty in Walking, and Age (in months). Depression status is recorded as a binary response variable with 16,250 depressed and 2608 non-depressed individuals; Categorical variables, Smoking, and Restless-sleep, take binary values; Difficulty-in-Walking, takes values 0,1,2, or 9; Frequent Vigorous Physical Activity has values 1,2,3,4, or 5; High Blood Pressure takes values 0,1,3, or 4; continuous variable, Age(in month), has a range from 224 to 1232 with a mean value of 801. For each individual, we include only for which all of the factors were recorded. In total, there are 18,858 people included in the analysis. Our clustering method correctly identified two clusters. AutoClass, however, detects nine clusters. Table 6 and Table 7 report the confusion matrix obtained by our method and AutoClass, respectively. In the Non-depressed group, our method correctly detected 86.75% of non-depressed individuals. In the depressed group, 30.98% of individuals are correctly detected. Since AutoClass finds 9 clusters, it is not feasible to make a fair comparison. Therefore, we describe the nine clusters declared by the AutoClass for the sake of completeness. Table 7 listed AutoClass clustered nine groups and the number of true depression and non-depression patients in each group. Since the depressed group is much smaller than the non-depression group, the information gain is a not suitable measure since the percentage of the depressed group is always small in comparison with the non-pressed group. The information gain for both our method and AutoClass is small and deemed not informative.

## 5. Discussion

We have proposed a clustering method that uses statistical distances and tests. Numerical results show that the proposed method outperforms the AutoClass algorithm based on classification rate and entropy measure. The proposed method does not em- ploy a global distance function or a parametric model. For future work, we could consider extending the proposed method to cluster spatial and temporal data.

## 6. Conclusions

Mixed data are prolific in scientific research such as business, engineering, life sciences, etc. It is imperative to develop a method that can cluster mixed data in order to discover true and significant underlying structures of a data set and classify observations into different subsets. We propose a non-parametric method that uses a local weighted chi-squared statistic to determine underlying clusters. The proposed algorithm does not require any model assumption for attributes or any expensive numerical optimization procedures. Because the proposed algorithm extracts clusters sequentially with one cluster at each iteration, it does not need any convergence criterion. The algorithm is terminated when all data points have been used and no more cluster centers can be detected. Consequently, our algorithm automatically produces the number of clusters, and the resulting partition is unique. When compared with the benchmark clustering algorithm for mixed data, AutoClass, we find that our algorithm outperforms AutoClass in various settings and produces similar accuracy in other settings.

## Figures and Tables

**Table 1:** Quantization levels. The means of F statistics, CR, and IG are obtained based on 500 replications.

Discretized Levels	Mean (F)	Mean (CR)	Mean (IGR)
5	630.1573	0.8302	0.7130
6	1523.4557	0.8455	0.7667
7	1722.3260	0.8227	0.6960
8	3223.9477	0.8635	0.7729
9	3916.3388	0.8816	0.7958
10	3708.5293	0.8682	0.7689
11	6444.7055	0.9085	0.8573
12	4778.9851	0.8893	0.8114
13	4912.8477	0.8907	0.8116
14	4262.3990	0.8907	0.8135
15	4000.3948	0.8879	0.8095
16	4234.9993	0.8863	0.7992
17	3549.8632	0.8787	0.7853
18	4042.0805	0.8785	0.7833
19	3657.4556	0.8768	0.7785
20	4303.8698	0.8872	0.8010

**Table 2:** Average CR and IGR with corresponding standard deviation for each method based on the simulated data of sample size 200 with 3 clusters; each cluster has sizes 130, 45, and 25, respectively. The mean values for each cluster are 2, 8, and 16 respectively. With the same set of means, the different variances, 0.25, 0.5, and 1 are compared. The number of replications is 500.

	AutoClass	Ours	AutoClass	Ours	AutoClass	Ours
	(Var = 0.25)		(Var = 0.5)		(Var = 1)	
CR Mean	0.6424	0.9556	0.6335	0.9292	0.6325	0.9370
CR Std	0.0021	0.0035	0.0015	0.0069	0.0015	0.0060
IGR Mean	1.0000	0.8923	1.0000	0.9085	1.0000	0.9148
IGR Std	<0.0001	0.0148	<0.0001	0.0094	<0.0001	0.0070

**Table 3:** Average CR and IGR with corresponding standard deviation for each method based on the simulated data of the sample size 1000 with 5 clusters; each cluster has sizes 500, 200, 100, 100, and 100, respectively. The mean values for each cluster are 2, 8, 16,20, and 35, respectively. With the same set of means, the different variances, 0.25, 0.5, and 1 are compared. The number of replications is 500.

	AutoClass	Our	AutoClass	Ours	AutoClass	Ours
	(Var = 0.25)		(Var = 0.5)		(Var = 1)	
CR Mean	0.5638	0.8747	0.5598	0.8792	0.5615	0.8777
CR Std	0.0016	0.0185	0.0015	0.0179	0.0014	0.0189
IGR Mean	0.7337	0.9228	0.7338	0.9174	0.7338	0.9235
IGR Std	<0.0001	0.0021	<0.0001	0.0049	<0.0001	0.0037

**Table 4:** Average CR and IGR with corresponding standard deviation for each method based on the simulated data of sample size 10,000 with 3 clusters; each cluster has sizes 5500, 3000, and 1500, respectively. Continuous data are from a multivariate t-distribution with degree freedom 5, 15, and 30, respectively. With the same set of means, the different variances, 0.25, 0.5, and 1 are compared. The number of replications is 100.

	AutoClass	Our	AutoClass	Ours	AutoClass	Ours
	(Var = 0.25)		(Var = 0.5)		(Var = 1)	
CR Mean	0.8120	0.9689	0.8231	0.9689	0.8202	0.9641
CR Std	0.0019	0.0031	0.0023	0.0031	0.0033	0.0034
IGR Mean	1.0000	0.9333	1.0000	0.9333	1.0000	0.9323
IGR Std	<0.0001	0.0067	<0.0001	0.0067	<0.0001	0.0048

**Table 5:** Two Real Data Results from two comparison methods. Heart data have 2 clusters with a sample size of 270 and Australian data has 2 clusters with a sample size of 690.

	Heart		Australian	
	AutoClass	Ours	AutoClass	Ours
CR	0.4444	0.8148	0.5217	0.7362
IGR	0.2754	0.6975	0.2761	0.8314
Number of clusters	5	2	7	2

**Table 6:** Confusion Matrix for our method.

		Our Method		
		Non-Depressed	Depressed	Total
True	Non-depressed	14,097	2153	16,250
	Depressed	1800	808	2608
	Total	15,897	2961	18,858

**Table 7:** Confusion Matrix for AutoClass.

		AutoClass									
		Clst1	Clst2	Clst3	Clst4	Clst5	Clst6	Clst7	Clst8	Clst9	Total
True	Non-depressed	3117	2362	1457	2039	1749	2032	1915	1201	378	16,250
	Depressed	216	217	781	335	461	158	144	223	73	2608
	Total	3333	2579	2238	2374	2210	2190	2059	1424	451	18,858

## Data Availability

1. Heart disease data: https://archive.ics.uci.edu/ml/datasets/Heart+Disease (accessed on 19 November 2022). 2. Australian Credit Approval: https://archive.ics.uci.edu/ml/datasets/statlog+(australian+credit+approval) (accessed on 19 November 2022). 3. RAND HRS data (Version O): https://hrsdata.isr.umich.edu/data-products/rand-hrs-archived-data-products (accessed on 19 November 2022).

## Abbreviations

The following abbreviations are used in this manuscript:

HD	Hamming Distance
DV	Distance Vector
UDV	Uniform Distance Vctor
CR	Classification Rate
IG	Informtion Gain
IGR	Inofrmaiton Gain Rate
